# Coupling Mass Spectrometry-Based “Omic” Sciences with Bioguided Processing to Unravel Milk's Hidden Bioactivities

**DOI:** 10.4172/2329-888X.1000104

**Published:** 2013-07-24

**Authors:** David C Dallas, Hyeyoung Lee, Annabelle Le Parc, Juliana Maria Leite Nobrega de Moura Bell, Daniela Barile

**Affiliations:** 1 Department of Food Science & Technology, University of California, One Shields Avenue, Davis, CA 95616, USA; 2 Foods for Health Institute, University of California, One Shields Avenue, Davis, CA 95616, USA

**Keywords:** Milk, Bioactive Molecules, Mass Spectrometry, Peptide, Glycan, Oligosaccharide, Glycolipid, Glycoprotein, Purification, Bioguided Processing

## Abstract

Many of milk's functional molecules could not be discovered until the right concordance of novel separation and analytical technologies were developed and applied. Many health-promoting components still await discovery due to technical challenges in their identification, isolation and testing. As new analytical technologies are assembled, new functional milk molecules will be discovered. Bovine milk is a source of a wide array of known bioactive compounds from a variety of molecular classes, including free glycans, lipids, glycolipids, peptides, proteins, glycoproteins, stem cells and microRNA. Because milk is such a complex mixture, when analyzed without fractionation or purification, many components mask the analytical signal of others, so some components cannot be detected. Modern analytics allow for the discovery and characterization of hundreds of novel milk compounds with high-resolution and high-accuracy. Liquid chromatography paired with electrospray ionization allows the separation of peptides, glycans and glycolipids for improved mass spectrometric detection. Target proteins and glycoproteins can now be purified from intact milk or other dairy streams by chromatography in order to better characterize these proteins for new bioactivities. The combination of advanced analytics with the new engineering capabilities will allow for high molecular resolution and separation techniques that can be scaled-up to semi-industrial and industrial scale for translation of lab-based discoveries. Bioguided analysis and design of dairy processing side streams will result in the transformation of waste into isolated functional ingredients to add value to dietary products.

## Introduction

Milk is the product of over 200 million years of mammalian evolution [1]. The constant evolutionary pressure on milk as the sole source of nourishment for mammalian infants has resulted in a remarkable model for how diet affects all aspects of development and health. As all components in milk are derived from the mother's own stores, milk likely contains only molecules that support the health of the infant. For this reason, milk is an incredible source for discovery of functional molecules. As a whole, milk supports growth, immune development, neurological development and microbial development of the infant [2].

Over the years, hundreds of compounds from a variety of molecular classes have been identified in milk. Novel milk molecules continue to be discovered. For each new molecular class, novel detection, separation and analysis methods must be established. The number of functions attributed to milk components is ever increasing. For example, individual classes of molecules in milk are known to limit pathogen growth [3,4], enhance commensal bacterial growth [5], modulate the immune system [6,7], decrease inflammation [8], and to have anti-viral [9] and anti-cancer activities [10]. Surprisingly, many of milk's functional compounds were discovered only recently, including naturally occurring peptides, oligosaccharides, stem cells, exosomes and microRNA. We hypothesize that many more functional molecules remain undiscovered in milk.

Milk's natural structural and compositional complexity precludes the use of a single modern analytical technique for analyzing all milk components. Rather, complete analysis of milk's components requires a combination of old and new tools. Many technological breakthroughs have made discovery of new milk molecules possible. These advances include improved separation science, high-resolution detection instrumentation, bioinformatic toolsets (like proteomic software) and genomics (as a tool to guide proteomics). Currently, toolsets exist for the detection of a wide array of milk lipids (lipidomics), some milk glycolipids, proteins (proteomics), naturally occurring peptides in milk (peptidomics) and free milk oligosaccharides (glycomics).

Now that these technologies exist, assessment of which dairy processing streams yield the highest concentrations of functional compounds is possible. Many processing streams at various steps of production need to be evaluated in detail for all carbohydrate structures: this labor-intensive process has driven the development of new methods and software to aid in automating the analyses. Different processing conditions can be analyzed to determine which produce highest bioactive compound yield and best preserve bioactivity.

After identification, there is a need for scaled-up production of each functional molecule/molecular class in order to test them for function in assays and clinical trials. Molecular classes that could be isolated from dairy streams include peptides, glycoproteins, glycolipids and free oligosaccharides. With successful separation of milk components and verification of their identicalness or similarity to human milk components, these compounds can serve as the basis for improved infant formulas or other therapeutic applications. Application of molecules shown to be functional to commercial products will increase the overall value of the dairy industry.

This review explores how the discoveries of novel components of milk have been made possible through advances in separation science and analytical chemistry, and how these molecules can be isolated at the industrial scale.

### Chromatography: an enabling step to reduce milk complexity

In order to clearly identify each molecular type by mass spectrometry, compounds must be purified from all contaminants and chromatographically separated. Without separation, molecules will compete for ionization. Molecules with low ionization efficiency will not be detected as their signals will be suppressed by other molecules that are easily ionized. Liquid chromatography separates molecules over time based on their affinity to a stationary phase. By separating molecules over time with chromatography, fewer molecules are introduced to the mass spectrometer at any one time, decreasing ion suppression and increasing the number of possible compounds detected. Liquid chromatography can be paired in-line with mass spectrometry via electrospray ionization. Nanoflow chromatography systems employ less sample and solvent than normal liquid chromatography and can be packed with a variety of solid phases allowing separation of peptides, glycans and other classes of molecules. Graphitized carbon columns can separate glycans down to the structural isomers level and thus enable structure-specific glycomics [11,12]. Peptides are typically separated with reverse-phase chromatography (non-polar stationary phase). Lipid chromatography can be performed with reverse phase and normal phases (eg. silica).

Modern liquid chromatography allows reproducible retention times that can aid in the identification of known molecules in the chromatogram. The mapping of compounds can be improved with higher reproducibility of column separation and sharper peak elutions (i.e., creation of new columns with higher theoretical plates). Retention times and mass can be used to identify specific known structures.

Compounds can also be fractionated on a preparative basis for further experimentation via flash liquid chromatography. For example, flash liquid chromatography with a porous graphitized carbon stationary phase was used to fractionate milligram quantities of glycan isomers from milk to generate standards that are not available commercially [13]. Fractionation of milligram quantities of sample allows testing of these different fractions in assays to discover specific functionality of each.

### Mass spectrometry: enabling molecular identifications in milk

**Figure 1:**
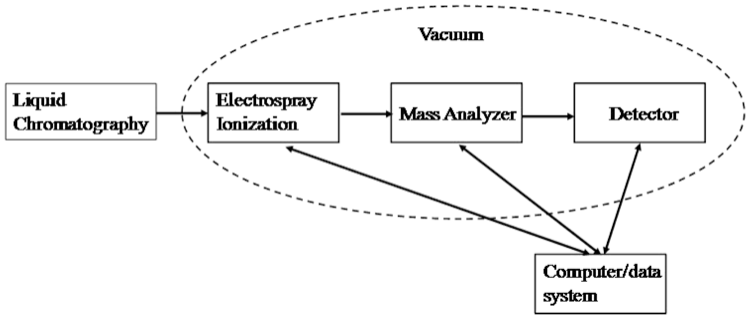
Basic components of mass spectrometers.

Mass spectrometric platforms are capable of detecting more compounds in a single run than other traditional analytical tools. The high mass accuracy available in modern mass analyzers dramatically reduces the number of possible analyte identities. With small molecules, this high precision measurement can lead to a single compositional possibility. A variety of mass spectrometers can be employed for detection of bioactive milk components (e.g. TOF, Fourier Transform Ion Cyclotron Resonance (FTICR)). These mass analyzers have high-resolution and are highly sensitive. Modern mass spectrometers allow detection of molecules at sub-femtomole concentrations. Figure 1 depicts the generic schema for mass spectrometers.

Advances in ionization technology were crucial to the use of mass spectrometry for milk component detection. Soft ionization techniques (which produce mainly molecular ions) like matrix-assisted laser desorption/ionization (MALDI) [14,15] and electrospray ionization (ESI) [16] are requisite for intact biomolecule analysis for nonvolatile, thermally unstable compounds such as peptides, glycans and glycolipids.

The mass of the intact molecule alone is often not enough to achieve unambiguous molecular assignment. To narrow down the list of potential molecular structures, unknown ions are sequentially isolated and fragmented. The fragments in these spectra allow for confirmation of one compositional identity. In some cases, tandem fragmentation can provide structural information.

Construction and application of molecular libraries allows streamlined, high-throughput identification of known molecules. Application of a molecular library within spectral analysis software (such as Agilent MassHunter) allows fast matching of ions to the library and computational extraction of each compound's abundance. These libraries typically include the mass, retention time and identity of the compound. More sophisticated libraries such as Agilent Personal Compound Database Library also employ representative MS/MS spectra for each compound to increase the certainty of the match. For these libraries, the MS/MS spectra of the unidentified compound must match, within a margin of error, the peaks in the reference spectra. These libraries allow for high-speed evaluation of samples from milk and dairy streams for their bioactive compound content.

### Potential uses of the technology to identify milk components

#### Peptidomics

Most milk proteins are non-functional when intact. Likely, their importance lies in the encrypted functional fragments that are released during digestion. A large number of naturally occurring peptides is known to exist in milk [17]. Milk peptides created by in vitro digestion have immunomodulatory, antimicrobial, opioid and nutritional functions [18].

Peptidomic analysis of milk's naturally occurring peptides is only now possible through a confluence of novel preparative isolation, analytical separation, analytical detection and computational techniques. Milk peptides can be isolated by first removing cream by centrifugation, then removing the large proteins via acid precipitation and finally removing the salts, lactose and oligosaccharides by preparative non-polar (C18) solid-phase extraction (SPE). Once isolated, peptides can be separated with a C18 analytical column and detected by a mass analyzer. Even low ppm precision intact mass measurement is not enough to identify the exact peptide sequence. Therefore, compounds can be sequentially isolated and fragmented. The fragment spectra can be analyzed for components of the peptide to provide evidence for which possible sequence is correct. As the mass spectrometer typically produces thousands of tandem spectra in an hour run, data must be processed via automated software. Data can be exported and the fragment spectra analyzed with proteomic software such as X!Tandem. Because milk's naturally occurring peptides are not cleaved in vitro by a single enzyme such as trypsin in most proteomic protocols, a search with a nonspecific enzymatic cleavage pattern must be performed. To narrow the possible peptide sequences, a milk protein-specific library can be employed as the basis for the search rather than the usual complete human proteome. The peptides identified from the automated analysis can be compiled as a library of known peptides with mass, retention time and a unique identity. This library can then be applied back to the mass spectrometer runs of the sample to extract peak volumes. Peak volumes can be compared between identical peptides across different sample sets.

Before the invention of soft ionization methods for mass spectrometry, including ESI and MALDI, peptides could not be analyzed by mass spectrometry as they are not volatile and are thermally labile. Without liquid chromatography (which is paired with ESI), the number of identifiable peptides would be greatly reduced. Without the low ppm mass accuracy measurements of modern mass spectrometers, peptides could not be identified. Without tandem fragmentation, peptides could not be sequenced with mass spectrometry. Advances in programming designed for proteomics were also essential to bringing about milk peptidomics. Simple modifications to the search parameters allowed application of these search algorithms to a new problem, which would have been impossible without this computational advance.

#### Glycolipidomics

Milk glycolipids exert important health effects, including prebiotic action, intestinal immune response modulation and acting as decoys for toxins [19]. Sialylated glycolipids-gangliosides-influence the microbiota that colonize the gut by supporting the growth of beneficial bacterial species, especially bifidobacteria. Infants fed a ganglioside-supplemented formula show lower count of intestinal pathogenic Escherichia coli than infants fed a standard formula [20]. Significant observations from animal studies show that inflamed intestinal mucosa has less sialylated gangliosides than healthy intestinal mucosa [21]. Accelerated catabolism of gangliosides in the intestine increases pro-inflammatory signaling, inflammatory markers and susceptibility to pathogens [22]. Dietary gangliosides can replace the mucosal gangliosides that are progressively degraded in inflammatory conditions [23]. Human and bovine milk gangliosides are similar to human intestinal membrane glycolipids as both contain GM3 (NeuAcα2-3Galβ1-4Glcβ-Cer) and GD3 (NeuAcα2-8NeuAcα2-3Galβ1-4Glcβ-Cer). Therefore, milk consumption might help restore proper ganglioside abundance and function in the intestine. This change could resolve inflammation, increase resistance to infection, and improve gut integrity to induce remission of gut inflammatory diseases like necrotizing enterocolitis, inflammatory bowel diseases and Crohn's disease.

**Figure 2:**
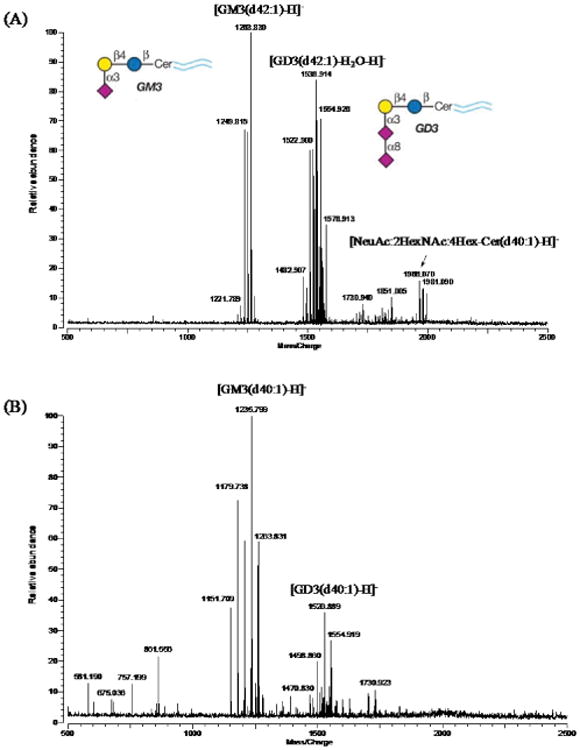
Representative MALDI-MS spectra of gangliosides from bovine and human milk. Negative mode MALDI MS spectrum of (A) bovine milk gangliosides and (B) human milk gangliosides. The inserts show the structures of GM3 and GD3 gangliosides.

The isolation and analysis of glycolipids are two of the most challenging analytical problems of lipidomics because these unique molecules have characteristics of both lipids (hydrophobic) and glycans (hydrophilic) and are not water-soluble. These physical properties make their separation exceptionally challenging. Because of their amphipathic nature and structural complexity, the classical methods for glycolipid analysis require extensive sample preparation, and provide only partial information on the oligosaccharide and ceramide portions. Recently, a method was described for the rapid and accurate profiling of human or bovine milk gangliosides with both glycan and lipid components identified using high resolution MALDI-FTICR mass spectrometry [24]. GM3 and GD3 are the most abundant gangliosides in the milks, and their ceramide structures were identified simultaneously (Figure 2). Measured accurate mass values combined with the Kendrick mass defect plot revealed each compound's oligosaccharide headgroup and the carbon distribution in ceramide backbone simply and rapidly.

Each ganglioside species can be quantified by liquid chromatography tandem mass spectrometry with the use of a triple quadrupole mass spectrometer in multiple reactions monitoring (MRM) mode. MRM provides a highly selective and very sensitive determination of gangliosides in a mixture, and it is quantitative when used with pertinent standards [25,26]. This quantitative method can be used to measure changes in gangliosides under various physiological and processing conditions.

#### Glycomics

Another class of milk components that has challenged researchers for years is free milk oligosaccharides. Recent research suggests that all mammalian milks contain a highly potent mixture of prebiotic glycans (oligosaccharides) with novel bioactivities that appear to protect and promote human health. The two recognized groups of milk oligosaccharides are common to most mammalian milk, including bovine milk, and are composed of neutral oligosaccharides that contain the monomers galactose, N-acetylglucosamine, fucose and a lactose core, and acidic oligosaccharides that contain the same monomers as well as N-acetylneuraminic acid (NeuAc), a type of sialic acid [27,28].

Because of their structural complexity, only bacteria that possess the necessary enzymes to cleave the specific glycosidic bonds that link the individual monosaccharides (fucose, sialic acid, N-acetylhexosamine (HexNAc), hexoses) together are able to utilize such complex oligosaccharides for growth. This genetic capability is thought to be part of the basis by which these beneficial bacteria out-compete pathogenic bacteria in the gut. In particular, the monosaccharide fucose and sialic acid appear to be crucial to the ability of oligosaccharides to selectively enrich beneficial bacteria. Sialylated HMO carrying one or more molecules of NeuAc are also thought to contribute to infant brain development by acting as a source of NeuAc for brain ganglioside assembly [29]. This hypothesis is supported by research showing higher I.Q. for breast-fed infants compared with formula-fed infants [30-32]. Bovine milk oligosaccharides (BMO) are similar in structure to HMO, which enhance the growth of commensal microbiota [33,34]. Bovine colostrum contains high levels of acidic sialylated oligosaccharides [33]. Bovine milk-based formulae contain these oligosaccharides only in trace amounts, whereas human milk contains 500-1000 mg/L of sialyl-oligosaccharides [35].

Many important applications await the isolation and characterization of the non- digestible oligosaccharides in milk. Unfortunately, at present there are no proven commercial sources for HMO-like compounds with high amounts of sialylation and fucosylation, thus the dairy industry is supplementing products with simpler oligosaccharides extracted from plants.

The structural complexity of oligosaccharides makes a single tool and a single method insufficient for their characterization. The major analytical techniques currently include mass spectrometry, chromatography such as high pH anion-exchange chromatography with pulsed amperometric detection and nuclear magnetic resonance spectroscopy. Several groups worldwide are working to complete the annotation of the human and bovine milk glycome [33,34,36-38]. Nano-LC-Q-TOF using graphitized carbon columns is emerging as the preferred method for oligosaccharide characterization because of the high sensitivity and reproducibility, and the minute amount of sample required, and because of the high-throughput that allowed construction of the first bioinformatic library of HMO containing over 75 fully annotated structures [38,39].

Commercial viability of large-scale extraction of bioactive molecules from agricultural commodities will depend on the richness of these molecules in the sources. Bovine milk contains only trace amounts of these valuable compounds, but many dairy side-streams are potentially rich in bioactive oligosaccharides. The recent discovery that whey permeate contains complex fucosylated and sialylated oligosaccharides analogous to the composition and structures of HMO demonstrates this stream as a promising source for industrial production of human-milk like oligosaccharides [40,41].

This discovery has led to collaborations with the US Dairy Industry to advance the research and development of these specific oligosaccharides as food ingredients. It appears that concentrating certain oligosaccharides from whey permeate can be a cost-effective process for the valorization of whey permeate into high quality and profitable novel dairy ingredients. Purified BMO extracted from cheese whey is currently being used in a human trial (ClinicalTrials.gov Protocol Record 264294).

**Figure 3:**
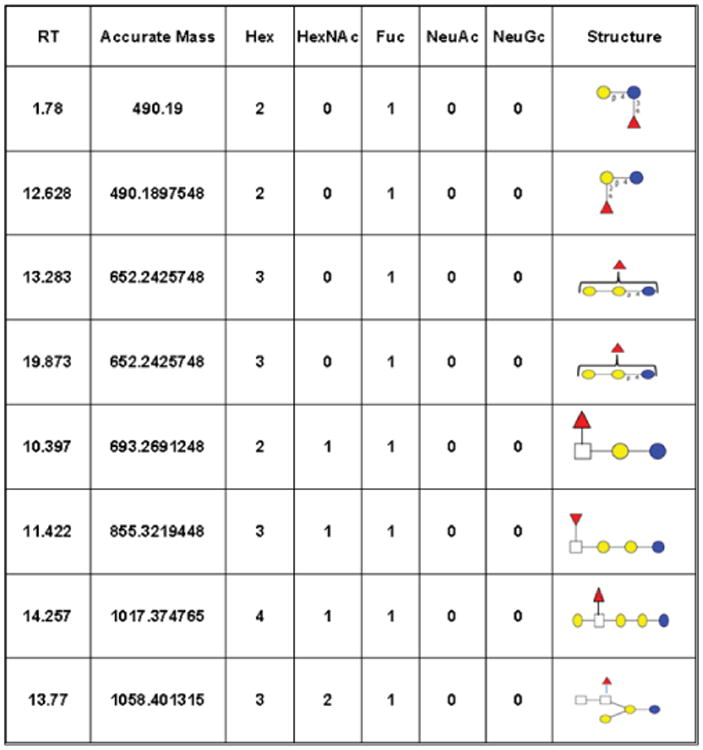
Example bioinformatic library for fucosylated BMO. Each entry includes retention time (RT), accurate mass, oligosaccharide composition (Hex (hexose-galactose in yellow and glucose in blue circles), HexNAc (*N*-acetylhexosamine in white squares) Fuc (fucose-red triangles), NeuAc, and NeuGc (*N*-glycolylneuraminic acid) and full/partial structure.

The use of whey permeates as a source of BMO is especially attractive due to the wide availability and low cost of this by-product compared with those of liquid bovine milk. There is an urgent need, however, to identify which dairy streams (whey permeate, mother liquor, etc.) have the highest concentration of bioactive glycans. The recent creation of a specific bioinformatic library of BMO that includes several novel fucosylated oligosaccharides will enable rapid identification of low abundance oligosaccharides in dairy streams [37]. Figure 3 presents a partial structure library detailing the most important fucosylated structures identified to date in bovine milk. Application of his library will facilitate structural identification in all dairy streams.

#### Glycoproteomics

Milk proteins are extensively post-translationally modified. Post-translational modifications (PTM) of milk proteins are the result of enzymatic processing of a polypeptide chain in the cell. Hundreds of PTM exist, including include glycosylation, phosphorylation, oxidation and deamidation. PTM influence compartmentalization, function, stability, structure, isoelectric point and proteolytic susceptibility of proteins [42,43]. Many milk proteins are decorated with long carbohydrate chains (glycosylation) during biosynthesis [44,45]. Glycans are linked to the protein through O-glycosidic or N-glycosidic bonds [46]. N-glycans are linked to the asparagine residues of proteins in the specific amino acid sequence Asn-XXX-Ser/Thr. The N-glycan core is composed of 2 N-acetylglucosamines (GlcNAc) and 3 mannoses. The different classes of N-glycan include high mannose (addition of mannose to the core), complex (addition of HexNAc to the core) and hybrid (combination of high mannose and complex). Additional glycan-like sialic acid and fucose can be added to the glycan structure. Protein-linked glycans are involved in multiple cellular mechanisms that contribute to the health and development of the infant [47]. The diverse protein-liked glycans may act as decoys for pathogens by competitively inhibiting their attachment to intestinal cell membrane glycans, thus reducing risk of infection [48]. The function of glycans varies based on the protein to which they are attached and the precise site of attachment [49]. Determining function of glycosylation on individual milk proteins remains a significant challenge. Whereas the primary structures of linear biomolecules such as nucleic acids and proteins may be elucidated simply by determining their sequence, glycans are branched, with both linkage and anomeric isomers, which significantly complicates their identification.

**Figure 4:**
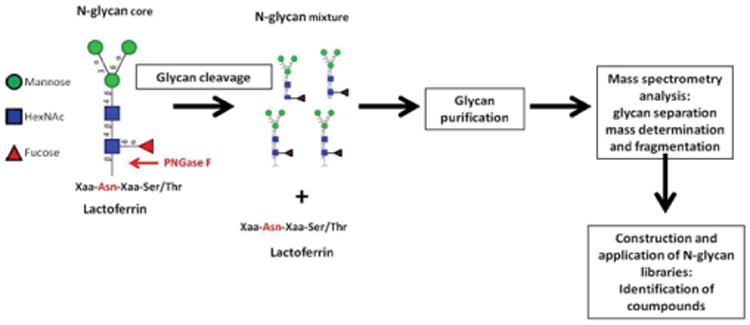
Schematic of enzymatic N-glycan release from protein and workflow for characterization of the released glycans.

Complete glycoprotein analysis requires determination of the sites of glycosylation and the glycan structures associated with each site. As complete glycoprotein analysis remains challenging, glycoproteins are typically analyzed either via proteocentric methods where the glycan is removed enzymatically and the protein identified or the glyco-centric method where the protein is removed and the glycans are identified [50]. Protein linked N-glycans can be released from the protein by a commercial enzyme, peptidyl-N-glycosidase F (PNGase F), which cleaves between the asparagine residue and the first GlcNAc. Figure 4 displays the specific of the mechanism of deglycosylation and the workflow for the characterization of released glycans

Released glycans can be purified by graphitized carbon chromatography. N-glycans are challenging to characterize because of their complexity and diversity. Highly sensitive analytical methods are required in order to separate and differentiate among the variety of glycan compositions and structures. Tandem mass spectrometry can help determine glycan structure. In some instances, a series of enzymatic cleavage steps with specific glycosidases is necessary for determining structure [51,52]. Once N-glycan structures are known, they can be compiled into libraries of known mass and retention time. These libraries can then be applied to new samples for fast identification of known molecules.

### Glycoprotein isolation

Milk glycoproteins are abundant both in the whey and in the casein micelles. Milk glycoproteins have a variety of health-promoting functions including antimicrobial, antiviral, antioxidant, antiinflammatory, immunomodulatory and anti-cancer actions [53-56]. These functional proteins can be applied as ingredients in functional foods, formulas and other therapeutics. Protein-linked glycans may help protect against microbial and viral attacks and help develop balanced intestinal microbiota [48,57-59]. Glycans impact structural and functional properties of proteins as well as the resistance to proteolysis and thermal stability [60,61].

**Figure 5:**
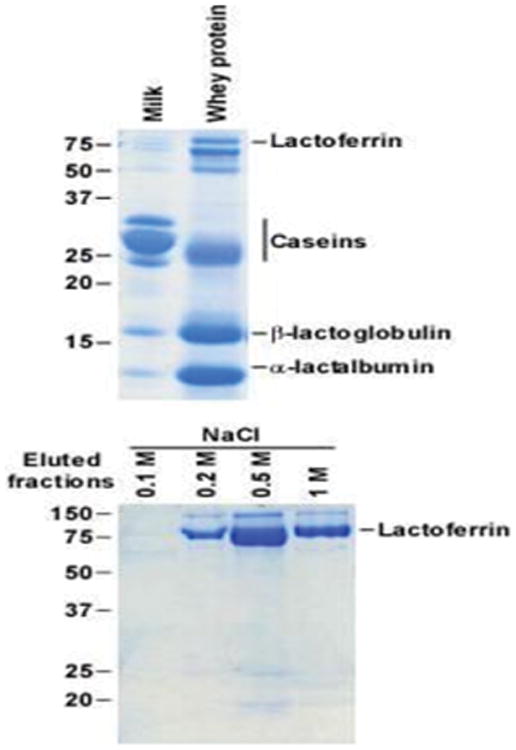
Electrophoretic gels demonstrate the sequential successful purification of target whey proteins (e.g. lactoferrin from the remaining whey proteins). Isolated fractions were analyzed on 12% SDS-PAGE after affinity chromatography.

Modern separation technology also allows the isolation of specific proteins and glycoproteins from milk and whey. For example, lactoferrin, which is one of the most abundant glycoproteins in many mammalian milks, can now be isolated from bovine whey in high purity via readily available chromatographic methods including ion-exchange chromatography [62] and affinity chromatography [48] (Figure 5). In these affinity resins, molecules (ligands) are immobilized on beads and packed in column. Novel chromatographic methods allow separation of glycoproteins based on their glycan composition [63]. For example, immobilized lectins selectively bind specific protein-linked glycans. The lectin concanavalin A, for instance, binds α-linked mannose and terminal glucose. These techniques allow high purity isolation of the protein of interest from a complex mixture. Optimization is required for each unique glycoprotein of interest. The quality of the purification can be monitored by electrophoretic methods and mass spectrometry. Isolated functional milk proteins can be isolated at industrial scale from milk and dairy streams to provide a functional ingredient for formula production, functional foods or therapeutic applications.

### Scale-up

The same techniques used for separation/purification of specific milk molecules at a laboratory scale can be scaled-up to industrial scale. Scaled-up isolation of milk molecules enables their use in functional testing and clinical trials. Some of the processes used to fractionate and purify dairy bioactive components (glycoproteins, peptides, glycolipids, and free glycans) at laboratory-scale include cream separation, ion-exchange and affinity chromatography, gel and capillary electrophoresis, selective precipitation, solid-phase extraction and membrane filtration. Except for cream separation and membrane applications, the commercial adoption of these techniques has been hindered by unacceptable economics at large-scale, operational complexity, low productivity, large effluent production, and product degradation associated with the use of high temperatures, pH, and salts [64,65].

Membrane separation is widely used in the dairy industry for isolation/fractionation of whey proteins [66]. Membranes with a wide array of pore sizes and different configurations are commercially available. Successive filtration steps with different pore size membranes allow the separation of numerous milk components according to their size. The increasing number of successful membrane applications in the dairy industry is a direct result of many advances in membrane technology such as increased membrane reproducibility and chemical resistance, development of modules that can be easily sanitized to comply with food grade requirements, development of commercial inorganic membranes (which allow use with wider temperature and pH ranges, have higher chemical resistance, extend operating lifetime, and have back-flushing capability), development of functionalized ion-exchange membranes (anion- and cation-exchange membranes, a possible alternative to using column chromatography) and improvement of membrane hydrodynamics system hardware [66,67]. Membrane-based separations can be effectively and economically implemented at large-scale, a requirement for most dairy applications [64]. Some of the membrane applications in dairy processing include the use of micro filtration membranes to remove milk fat and bacteria, ultrafiltration membranes to recover and/or fractionate whey proteins and casein micelles from milk, and the combination of ultra-and nanofiltration to recover oligosaccharides [64,66,68].

Currently, chromatography scale-up techniques are expensive because of the cost of their solid phase. However, innovations are helping to make these techniques increasingly economically feasible. For example, research to improve the feasibility of large-scale chromatography through regeneration and reuse of both solid-phase materials and solvents are ongoing [69]. Recent developments in industrial-scale bio-chromatography columns such as the development of new column designs that promote optimal performance with any resin at any scale and that are easy and safe to handle suggest that larger scale isolation of glycoproteins is feasible.

Separation of proteins with similar size but different isoelectric points can be achieved by the use of charged membranes at specific pH and ionic strength [70]. This combination has the potential to overcome a major impediment for protein fractionation-the permeability-selectivity trade-off-and expand the use of membrane filtration in downstream processes where chromatography was previously necessary. One recent development in membrane-based technologies-the coupling of imposed electric fields and pressure-driven filtration [71,72] has potential to improve the separation and purification of milk bioactives. The application of an electric field across a membrane during filtration can enhance the fractionation of negatively or positively charged molecules. Like-charged large molecules are repelled from the membrane surface, which allows for increased transmission of smaller molecules through the membrane [71]. In comparison to normal membrane filtration, electric field-enhanced membrane filtration applied to proteins and peptides has been shown to reduce membrane fouling and, therefore, improve permeate flux and fractionation selectivity [71,72].

**Figure 6:**
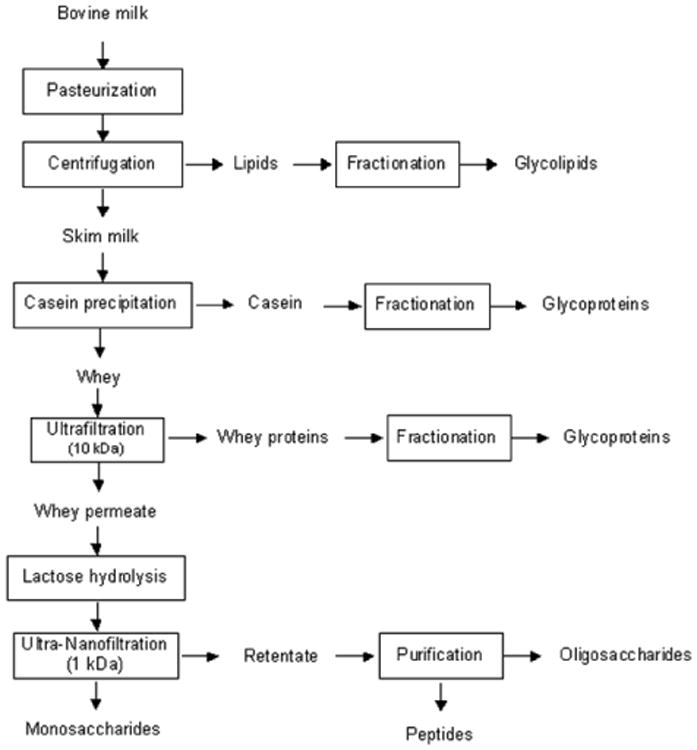
Schematic of potential pathways to maximize the recovery of milk bioactive components.

The global production of liquid whey amounted to 186 million metric tons in 2008 [73] and it will continue to grow, along with cheese production. For environmental reasons, whey is no longer discharged into rivers because of its high biochemical and chemical oxygen demand (index of pollution). This stream, once considered waste by the dairy industry, is now known to contain a variety of functional components including glycoproteins, oligosaccharides and naturally occurring peptides. Whey proteins are now isolated by membrane filtration, but leave behind whey permeate that still contains the oligosaccharides and naturally occurring peptides. This whey permeate is currently disposed of by the manufacturer. The functional molecules in these dairy streams should be captured for further use. Current technologies allow for the capture of these components. A general schematic for industrial-scale isolation of functional milk components such as glycoproteins, glycolipids, peptides and oligosaccharides from bovine milk is presented in Figure 6.

### Glycolipid isolation

Lipids can be isolated by either centrifugation/cream separator or membrane microfiltration (pore size range 0.10-0.5 μm) [66]. Solvent extraction, which is commonly used by the food industry, can be used to isolate glycolipids from neutral lipids (e.g. triglycerides). Then, reverse-phase chromatography can be employed at large-scale to remove other components like oligosaccharides, peptides and lactose.

## Whey Protein Isolation

The dairy industry employs ultrafiltration technology extensively for recovery of soluble whey protein concentrates from whey [66]. The development of robust membrane systems, different membrane configurations and continuous operation using multi-stages associated with the use of diafiltration (addition of solvent to wash micro-solutes from the retentate, therefore increasing the purity of the macromolecules retained have contributed to the success of this technology in the dairy industry [66]. Ultrafiltration of whey proteins generates two streams, a protein-rich retentate and a carbohydrate-rich permeate (oligosaccharides, lactose).

## Glycoprotein Isolation

Glycoproteins from whey protein concentrate can be isolated from the whey or casein protein fractions at industrial scale by chromatography. For example, lactoferrin can be isolated at industrial scale by ion-exchange chromatography [74]. Bovine lactoferrin is produced from whey cheese or raw milk by dairy industries for use in bovine milk-based infant formula for health claims.

### Oligosaccharide isolation

A major challenge for recovery of oligosaccharides from dairy streams is to simultaneously enrich the oligosaccharide concentration and deplete the content of lactose and other simple sugars that lack bioactive functionality. Lactose removal is important for bioactivity studies because it is the most abundant carbohydrate in whey and can confound studies on the biological activity of oligosaccharides. An inexpensive and scalable approach to recover biologically active oligosaccharides from milk was developed. This approach relies on the combination of enzymatic hydrolysis of lactose, a two-step filtration (ultra- and nano-filtration), followed by diafiltration of the nano-filtration retentate in order to remove the monosaccharides, thus enriching the retentate oligosaccharide content. With this approach, more than 50% of human milk's free oligosaccharides were recovered.

Further processing, enrichment and purification methods (Figure 6) are being investigated at pilot scale to optimize recovery of oligosaccharides from agricultural streams in a way that maximizes the concentration of specific structures present in human milk and depletes structures not present in human milk.

### Peptide isolation

Naturally occurring milk peptides will remain in the whey permeate with the oligosaccharides and lactose after ultrafiltration (10-30 kDa). Separation of these peptides from this solution has not been performed yet. This separation would require the use of large-scale chromatography, possibly with reverse- or normal-phases.

The development of feasibly priced, fully scalable fractionation and purification technologies is essential to enable large-scale production of purified bioactive milk compounds for their utilization in clinical trials. These isolated components can be employed as food products and supplements and may have significant health benefits for human nutrition.

## Conclusion

Advances in separation and analytical technologies are allowing discovery of hundreds of novel, functional milk molecules. The dairy industry has benefitted and will continue to benefit from the discovery of novel functional molecules in milk.

High-resolution mass spectrometry, molecular libraries and minute sample concentration requirements enable high-throughput investigation of different milk sources, including different dairy processing streams, breeds and species, for highest concentration of these compounds. The effects of genetic polymorphisms, environmental conditions, feeding strategies and lactation stage on the abundances of functional components will need to be investigated. Some species of lactating farm animals (cows, goats, sheep, etc.) and some breeds within those species are likely to have different levels of certain components more similar to those in human milk. Characterization of such a complexity is crucial for identification of viable sources for purification. For example, a recent study suggested that Danish Jersey milk is richer in bioactive (fucosylated) oligosaccharides than Danish Holstein-Friesian milk, and thus may have greater health-promoting benefits for consumers and may be a richer source for fucosylated oligosaccharide isolation [40]. Each species evolved with different physiological needs, which means each species' milk will have a slightly different repertoire of functional compounds for protecting and nourishing their offspring. Therefore, novel components in dairy animal milk that are lacking in human milk may nonetheless be employed in human therapeutics.

Assessing all of these elements was impossible a decade ago. However, the confluence of modern “omics” sciences and high-throughput mass spectrometry now makes this task is imminently achievable.

The scale-up of laboratory-based processing and purification techniques to industrial level at feasible cost is essential to achieve commercial production of these dairy-derived functional components as health-enhancing ingredients. By identifying functional components in dairy waste streams, this science will allow the dairy industry to improve their profitability. Partnerships between academia and industry will catalyze breakthroughs in isolation and fractionation of these functional ingredients from dairy streams. This will help bridge the gap from laboratory-based research to applications and accelerate the translation of these components to the marketplace.

## Abbreviations

BMO: Bovine Milk Oligosaccharides
HMO: Human Milk Oligosaccharides
MALDI: Matrix-assisted Laser Desorption/Ionization
ESI: Electrospray Ionization
Q-TOF: Quadrupole Time-of-flight Mass Spectrometer
Nano-LC-Q-TOF: Nanospray Liquid Chromatography Q-TOF
FTICR: Fourier Transform Ion Cyclotron Resonance
SPE: Solid-phase Extraction
MRM: Multiple Reaction Monitoring
NeuAc: N-acetylneuraminic acid
